# Rare Causes of Abdominal Pain

**DOI:** 10.1016/j.acepjo.2025.100096

**Published:** 2025-03-08

**Authors:** Wei Zheng, Xiaoyan Yu, Suying Wu

**Affiliations:** Department of Radiology, The First Hospital of Putian City, Putian, Fujian, China

**Keywords:** Chilaiditi syndrome, abdominal pain

## 1 Case Presentation

A 61-year-old man presented with a 6-hour history of persistent dull pain in the right upper abdomen, gradually worsening over time, accompanied by nausea and vomiting of gastric contents. The pain did not radiate, was unrelated to position changes, and had no associated fever, chills, abdominal distension, diarrhea, or bowel obstruction. Physical examination revealed mild right upper abdominal tenderness without rebound tenderness, with normal bowel sounds. Laboratory tests were unremarkable, and an abdominal computed tomography scan was performed for further evaluation with results shown in Figure A and B. What is the cause of the abdominal pain and the patient’s diagnosis?

**Figure:**
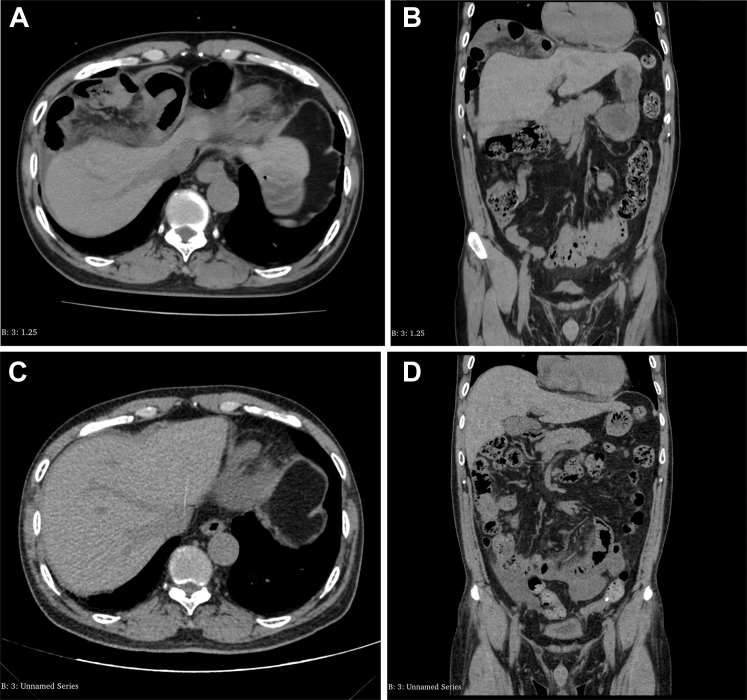
A, Axial view of the patient’s abdomen on computed tomography (CT) imaging. B, Coronal view of the patient’s abdomen on CT imaging. C, Axial view of the patient’s abdomen on CT imaging. D, Coronal view of the patient’s abdomen on CT imaging.

## 2 Diagnosis: Chilaiditi Syndrome

After conservative treatment during hospitalization, the patient’s symptoms improved, and follow-up computed tomography scans are shown in Figure C and D. Chilaiditi sign describes the positioning of the colon between the liver and diaphragm on imaging and is typically asymptomatic. When symptomatic, it is termed Chilaiditi syndrome.^1^ This syndrome was first reported in 1910 by Greek radiologist Demetrius Chilaiditi.^2^ The incidence of Chilaiditi syndrome is approximately 0.025% to 0.28%,^3^ with a higher prevalence in elderly men. The exact etiology of Chilaiditi syndrome remains unclear.^4^ Research by Inagaki and Ebata^1^ indicates that the interposed organ is most commonly the colon (93.1%), with the small intestine accounting for approximately (6%) of cases, with small bowel interposition being more likely to cause an acute abdomen. Most cases are asymptomatic. Symptomatic patients may need rest, fluids, decompression, or surgery for obstructions or ischemia.^5^

## Funding and Support

By *JACEP Open* policy, all authors are required to disclose any and all commercial, financial, and other relationships in any way related to the subject of this article as per ICMJE conflict of interest guidelines (see www.icmje.org). The authors have stated that no such relationships exist.

## Conflict of Interest

All authors have affirmed they have no conflicts of interest to declare.
